# Role of Fecal Microbiota Transplantation in Reducing Clostridioides difficile Infection-Associated Morbidity and Mortality: A Systematic Review

**DOI:** 10.7759/cureus.28402

**Published:** 2022-08-25

**Authors:** Adarsh Srinivas Ramesh, Carlos Munoz Tello, Dawood Jamil, Hadrian Hoang-Vu Tran, Mafaz Mansoor, Samia Rauf Butt, Travis Satnarine, Pranuthi Ratna, Aditi Sarker, Safeera Khan

**Affiliations:** 1 Internal Medicine, California Institute of Behavioral Neurosciences & Psychology, Fairfield, USA

**Keywords:** fecal microbiota transplantation (fmt), nosocomial infection, fecal transplantation, antibiotics associated diarrhea, recurrent clostridium difficile infection, fecal microbiota transplant, pseudomembranous colitis, toxic megacolon, fecal microbiota transplantation in clostridium difficile infection, clostridium difficile infection treatment

## Abstract

*Clostridioides difficile (C. difficile)* is a gram-positive, anaerobic, spore-forming bacterium that produces toxins A and B, disrupting the intestinal brush border and resulting in severe diarrhea. The most common causes of infection include prolonged antibiotic use, proton pump inhibitors (PPIs), and long-term hospitalization resulting in complications such as pseudomembranous colitis and toxic megacolon. This systematic review aims to consider fecal microbiota transplantation (FMT) as an early treatment modality in *C. difficile* infection to prevent complications and reduce related morbidity and mortality. We systematically screened three databases using regular keywords such as “fecal microbiota transplantation,” “*C. difficile*,” “pseudomembranous colitis,” and “toxic megacolon” and Medical Subject Headings (MeSH) terms. We applied the inclusion and exclusion criteria and performed a thorough quality appraisal using standardized checklists. We were finally left with 10 articles, including seven case reports, one case series, and two observational studies. Questions remain as to the route of administration of FMT, timing, safety, availability, and the number of sittings required. More randomized controlled trials are needed to address all these questions and to assess the safety of FMT. We believe the role of FMT is very important as it can prevent *C. difficile* related complications and would be an ideal treatment option in a population group that is often unfit for surgical management.

## Introduction and background

*Clostridioides difficile (C. difficile)* is reported to cause almost half a million infections in the United States yearly, with one in 11 people over the age of 65 diagnosed with a healthcare-associated *C. difficile *infection dying within one month [1]. Inappropriate antibiotic use in hospitalized patients, especially patients in the intensive care unit (ICU), is the leading cause of *C. difficile* infection resulting in more than 29,000 deaths every year [1-3]. Other major risk factors include proton pump inhibitor (PPI) use, history of treatment in the intensive care unit, recent surgery, immunocompromised states, and obesity. From acute care facilities alone, *C. difficile* is believed to incur up to 2.4 billion dollars every year in healthcare costs [2].

*C. difficile* is a gram-positive, spore-forming anaerobic bacterium with a wide spectrum of clinical presentations ranging from mild diarrhea to pseudomembranous colitis, ileus, toxic megacolon, and bowel perforation leading to peritonitis, sepsis, and shock [4]. Fulminant *C. difficile* leads to toxic megacolon, has a mortality rate of almost 50%, and in some cases is found to be refractory to medical management with drugs such as metronidazole, vancomycin, and fidaxomicin [5]. 

Fecal microbiota transplant (FMT) is an ideal treatment option in recurrent, refractory, and fulminant *C. difficile* infection, achieving cure in >90% of patients [6]. FMT is also useful in surgically unfit patients where colectomy is not possible and even in cases where FMT is not completely effective, it may quickly stabilize patients for surgery. Multiple sittings are required where 100 milliliters of the stool are administered with saline [5,7]. The latest research suggests that FMT via colonoscopy may be slowly replaced by the oral consumption of laboratory-designed frozen oral capsulized preparations [8]. FMT also has a wide array of applications other than in *C. difficile infection*, including in inflammatory and irritable bowel disease, diabetes and metabolic syndrome, refractory diarrhea, and even in neurological diseases like Parkinson’s disease and multiple sclerosis in addition to neuropsychiatric conditions like autism spectrum disorders [3,8]. 

One major concern in using FMT is the delivery of the feces. Colonoscopic delivery and prior bowel preparation risk perforation, especially in toxic megacolon patients, and nasogastric delivery may not be possible due to associated poor bowel motility [6]. Other problems include an insufficient screening of donors leading to other infections, including the coronavirus, and the lack of availability of healthy donors [3,8]. Although many studies have proved FMT to be a productive and safe mode of treatment for recurrent *C. difficile*, studies of its use in ICU patients to prevent *C. difficile* progression to toxic megacolon and reduce associated morbidity/mortality are inconclusive and yet to be explored [5]. There is also limited data on proper patient preparation, method of administration, and the timing of FMT [9]. This review aims to find evidence that FMT can be routinely used as a reliable treatment option that can significantly reduce morbidity and mortality associated with *C. difficile* and its complications.

## Review

Methods

We strictly followed Preferred Reporting Items for Systematic Reviews and Meta-analyses (PRISMA) 2020 guidelines to review and report our methods and results for this systematic review [10].

Search Sources and Strategy

PubMed, PubMed Central (PMC), ScienceDirect, and Google Scholar were the databases used. Regular keywords such as “fecal microbiota transplantation,” “*C. difficile*,” “pseudomembranous colitis,” and “toxic megacolon” were used independently and in combination with each other using Boolean operators to further specify our literature search. 

Medical Subject Headings (MeSH) strategy was also used in PubMed. After eliminating duplicates, the papers were first screened by going through titles and abstracts and selecting relevant articles, followed by full-text papers. Inclusion and exclusion criteria were applied, and the quality appraisal was done for the remaining 19 papers.

Data Extraction

The data retrieval and review were done independently by two separate researchers. If there were any differences of opinion, we would discuss them among ourselves to see whether the data met the requirements for eligibility. A third researcher was consulted if a decision could not be made.

Inclusion and Exclusion Criteria

The 19 articles selected for review were limited to papers published from January 2012 to December 2021 and were only in English. Papers based on animal studies, grey literature, and unpublished literature were excluded from the review. 

Assessment of Study Quality 

Since we have included multiple study designs, various quality appraisal tools such as Joanna Briggs Institute (JBI) checklist for case reports and case series were used, with articles being included if they scored eight or higher [11]. Newcastle Ottawa tool was used for observational studies, with articles being included if they scored 10 or higher. Table 1 summarises the results of the quality assessment [11].

**Table 1:** Joanna Briggs Institute checklist for case reports 1) Were the patient's demographic characteristics clearly described? 2) Was the patient's history clearly described and presented as a timeline? 3) Was the current clinical condition of the patient on presentation clearly described? 4) Were diagnostic tests or assessment methods and the results clearly described? 5) Was the intervention(s) or treatment procedure(s) clearly described? 6) Was the post-intervention clinical condition clearly described? 7) Were adverse events (harms) or unanticipated events identified and described? 8) Does the case report provide takeaway lessons? Y: yes, N: no, U: unclear, +: include

1	2	3	4	5	6	7	8	OUTCOME	AUTHOR
Y	Y	Y	Y	Y	Y	Y	Y	+	Gweon et al. [5]
Y	Y	Y	Y	Y	Y	N	U	+	Konturek et al. [12]
Y	Y	Y	Y	Y	Y	N	Y	+	Benech et al. [13]
Y	Y	Y	Y	Y	Y	Y	Y	+	Stein et al. [14]
Y	Y	Y	Y	Y	Y	Y	Y	+	Mankal et al. [15]
Y	Y	Y	Y	Y	Y	Y	Y	+	Huang et al. [16]
Y	Y	Y	Y	Y	Y	Y	Y	+	Yu et al. [17]

Results

**Figure 1:**
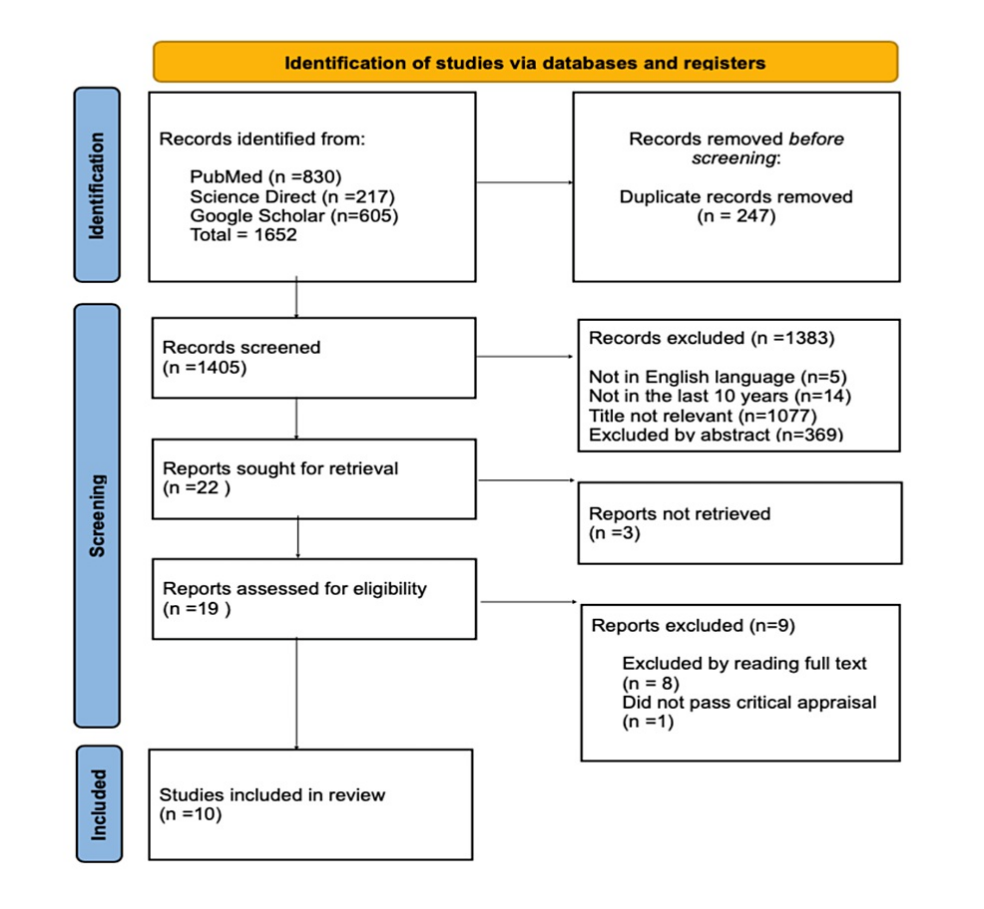
PRISMA 2020 flow diagram PRISMA: Preferred Reporting Items for Systematic Reviews and Meta-Analyses

This systematic review was conducted following the Preferred Reporting Items for Systematic Reviews and Meta-Analyses (PRISMA) guidelines. Our PRISMA flow diagram is shown in Figure 1 [10].

A total of 1652 articles were found using the MeSH strategy and regular keywords in multiple databases. Of the 1652 articles, 830 were from PubMed, 217 from ScienceDirect, and 605 from Google Scholar. Two hundred forty-seven duplicate articles were removed. Out of the remaining 1405 articles, 1383 were discarded based on the title, abstract, and application inclusion and exclusion criteria. The remaining papers were reviewed, and the full text of three out of the 22 articles was not available. After doing the quality appraisal, 10 papers were selected, out of which there were seven case reports, two observational retrospective cohort studies, and one case series. A brief description of the studies included in the review is shown in Table 2.

**Table 2:** Data extraction table FMT: fecal microbiota transplantation, *C. difficile*: *Clostridioides difficile*, DMD: Duchenne muscular dystrophy

Author	Year of Publication	Study	Results/Conclusion
Bamba et al. [3]	2019	Fecal microbiota transplantation in Japanese patients with refractory *C. difficile* infection.	This case series presented four patients with refractory *C. difficile* infection successfully treated with a single FMT procedure, with none of the patients relapsing after three months.
Gweon et al. [5]	2015	Toxic megacolon caused by *C. difficile* infection, treated with FMT.	FMT is a treatment option for *C. difficile* infection, but the position of colonoscopy can worsen hemodynamic status, and there is a risk of bowel perforation.
Cheng et al. [7]	2020	FMT decreases mortality in patients with refractory severe or fulminant *C. difficile* infection.	FMT was associated with significant decreases in *C. difficile* related mortality in patients with refractory disease. Therefore, it should be considered in patients who fail antimicrobial therapy and would otherwise be referred for surgery.
Tixier et al. [12]	2019	FMT decreases mortality in severe and fulminant *C. difficile *infection in critically ill patients.	FMT resulted in a 77% decrease in mortality in hospitalized patients with fulminant *C. difficile* infection, especially when started early.
Konturek et al. [13]	2016	FMT and fidaxomicin for severe pseudomembranous *C. difficile* colitis.	After ineffective treatment with vancomycin and metronidazole, a combination of FMT and fidaxomicin healed *C. difficile* colitis.
Benech et al. [14]	2020	Septic shock due to refractory severe *C. difficile* colitis rapidly resolving after fecal microbiota transplantation.	This case report showed that FMT could be an alternative to colectomy for complicated *C. difficile* infection, even in patients with hemodynamic instability, and initiation of FMT resulted in improvement.
Stein et al. [15]	2015	Toxic Megacolon from *C. difficile* infection successfully treated with FMT.	Two cases of toxic megacolon requiring colectomy, with computed tomography showing colonic dilation in both patients, were cured with FMT after failing antibiotic therapy.
Mankal et al. [16]	2017	Combination of fidaxomicin and FMT for severe *C. difficile* colitis.	This case report described a patient with *C. difficile* colitis with unstable vitals. Standard medical therapy failed, following which FMT and fidaxomicin were started, resulting in a cure.
Huang et al. [17]	2020	Treatment of pseudomembranous colitis with fecal microbiota transplantation.	The patient suffered from gastrointestinal ischemia due to a cardiac arrest resulting in *C. difficile* infection, which was refractory to broad-spectrum antibiotics, treated successfully with multiple rounds of FMT.
Yu et al. [18]	2016	FMT as a treatment for *C. difficile* related toxic megacolon in a patient with DMD.	DMD results in slower gastrointestinal motility and therefore predisposes to *C. difficile* infection, treated successfully with FMT.

Discussion

Risk Factors and Pathophysiology

*C. difficile* is a spore-forming, gram-positive anaerobic organism that can be community-acquired through fecal-oral contamination or hospital-acquired [4]. The use of antibiotics and PPIs are the most common means of acquiring *C. difficile* infection [5]. Most hospitalized patients, especially in the intensive care units, receive a significant number of medications for various ailments. The most commonly associated antibiotics are clindamycin, penicillin, cephalosporins, and fluoroquinolones [12]. Antibiotics destroy healthy normal gut flora, resulting in gut dysbiosis. This is where FMT proves to be an important treatment modality in restoring normal gut flora [13]. Benech et al. described a patient with a bone infection taking clindamycin for three months resulting in *C. difficile* infection presenting with intractable diarrhea [14]. 

Another reason is chronic PPI use, as it reduces the acidic pH of the stomach, which protects against a multitude of microorganisms. Patients with atherosclerotic cardiovascular disease who have reduced blood flow can suffer from hypoxia of the bowel, increasing the risk of infection. Gut motility also plays an important role in *C. difficile* infection. Patients with diabetes, scleroderma, and hypothyroidism are more prone to *C. difficile* infection [12]. Other conditions that slow gastrointestinal motility include surgery, causing postoperative ileus, and drugs such as narcotics and anticholinergics. Frequent enemas are also implicated as they result in eliminating the normal gut flora [12]. Inflammatory bowel disease is also associated with *C. difficle* infection, especially with ulcerative colitis resulting in more severe disease and complications such as toxic megacolon [4,15]. 

*C. difficile* is said to be a ubiquitous organism, producing toxin A and toxin B, both of which are exotoxins [15]. These exotoxins are believed to increase the permeability of the brush border enterocytes by disrupting tight junctions, resulting in diarrhea [13,16]. North American pulsed-field gel electrophoresis type 1 (NAP1), restriction endonuclease analysis type B1, and polymerase chain reaction ribotype 027, collectively known as NAP1/B1/027 strain showed a much higher recurrence rate than other strains. Many non-toxigenic strains only result in asymptomatic carriers [16]. 

Clinical Spectrum of C. difficile Infection

The clinical presentation of *C.difficile* infection is a spectrum and can vary from mild disease to pseudomembranous colitis and death [4]. In an otherwise healthy patient or patient affected with non-toxigenic strains, *C.difficile* can present with diarrhea and fever and resolve without any major complications [13]. Mankal et al. described a patient with lung cancer having pneumonia treated with antibiotics who returned with hemodynamic instability and fever with imaging showing thickening of the colon followed by multiple episodes of watery stool confirming recurrent *C. difficile* infection [16]. In a different case report, Huang et al. described a 16-year-old girl who experienced a cardiac arrest and needed cardiopulmonary resuscitation, which resulted in protracted gastrointestinal ischemia and *C. difficile* infection. Long-term antibiotic use was another important element. She presented with intractable diarrhea, fever, hematochezia, electrolyte imbalances, peritonitis, and sepsis [17]. 

Multiple case reports also showed that toxic megacolon is one of the serious complications of *C. difficile* infection [5,15,18]. A 26-year-old man developed severe diarrhea, abdominal pain, and fever after being treated with ciprofloxacin for a urinary tract infection. Stool polymerase chain reaction (PCR) was positive for *C. difficile*, and the patient was started on empiric vancomycin with no response. CT showed thickened and dilated colonic walls with fat stranding, confirming the diagnosis of toxic megacolon [18]. Another study described a case of toxic megacolon with 12cm dilatation of the right colon with elevated lactate and white cell count requiring right colectomy [15]. A further sequence of events includes bowel perforation leading to peritonitis, sepsis, and shock [4]. Toxic megacolon can lead to perforation in six to eight percent of patients and has a mortality rate of 30-80 percent [5,18]. 

Management of C. difficile Infection

One study mentioned starting with 500 milligrams intravenous metronidazole three times a day and 125 milligrams oral vancomycin four times a day for 10 days as initial treatment [5]. Another study provides the alternative view that antibiotics can further reduce intestinal flora, worsening the infection [17]. Taking both points of view into account, an ideal choice of antibiotic would be fidaxomicin. This macrolide antibiotic has little to no effect on the normal colonic flora and is efficacious against *C. difficile* infection, especially when combined with FMT. Vancomycin or fidaxomicin, along with FMT, can increase the cure rate by up to 90 percent when done sequentially [7,13]. Vancomycin can also be given rectally in patients with reduced gastrointestinal motility [16]. Metronidazole is also a treatment option, but many studies preferred vancomycin over metronidazole as they believed the former had higher rates of cure for *C. difficile* related colitis [17]. Other possible pharmacological options include rifaximin, tigecycline, nitazoxanide, and teicoplanin. Along the lines of promoting normal gut flora, probiotics, either naturally occurring (yogurt, fermented milk, kimchi, pickles) or capsules containing *Bifidobacterium*, *Saccharomyces boulardii*, and *Lactobacillus* can be used as an additional treatment modality [18]. 

The indications for surgery are toxic megacolon, colonic perforation, and peritonitis, all of which have a very high mortality risk [19]. Usually, patients with *C. difficile* infection-related complications requiring surgery are poor surgical candidates with multiple comorbidities or are in the intensive care unit with hemodynamic instability and therefore not fit for surgery [5,16]. FMT provides a safer alternative to colectomy in toxic megacolon patients, a procedure with high mortality rates [16]. In a study conducted in 2016, a patient with toxic megacolon underwent laparoscopic colectomy with loop ileostomy as the patient had pre-existing ileus, and the vancomycin lavage was done through the ileostomy tube [18]. Despite surgery, the patient continued to have diarrhea, and hence the option of FMT was considered [17].

Role of Fecal Microbiota Transplantation

In a study published by Benech et al., after failed antibiotic therapy and the patient being unfit for surgery, it was decided to start FMT with 83 grams of feces diluted in normal saline, resulting in resolution of diarrhea within six hours and by day seven, negative stool PCR for *C. difficile* [14]. Usually, one part of the stool is blended with three saline parts [5]. Before FMT can be done, screening of the donor feces is required. A case report describes a patient’s sister as a donor where her stool was screened for *C. difficile*, ova, parasites, human immunodeficiency virus (HIV), hepatitis A, B, and C, *Helicobacter pylori*, syphilis, cryptosporidia, microspora, and multiple other microorganisms. The stool was administered colonoscopically within two hours of collecting the donor stool [18]. A different study needed a donor who hadn't taken antibiotics in the previous year and hadn't received chemotherapy [5]. Sometimes FMT requires multiple sittings to achieve cure. A study conducted by Huang et al. required the patient to have four fecal microbiota transplants to resolve the infection completely [17]. 

The route of FMT depends on the patient’s clinical status. A study described using the rectal approach via the channel of the colonoscope in order to take biopsies, as the patient had poor intestinal motility from pre-existing ileus. In contrast, another study delivered stool in the oral route via a nasogastric tube to a patient with toxic megacolon, to reduce of risk perforation [14,4]. Duodenal infusion has the advantage of a longer retention time. It can be used in semiconscious patients as feces may not be retained in the bowel in the colonoscopic approach [5]. FMT via the nasogastric approach has a risk of aspiration leading to pneumonia [19]. Stein et al. used a combination technique of delivery, where 700 milliliters of feces were administered to the jejunum through push enteroscopy and 300 milliliters through flexible sigmoidoscopy to the descending colon [15]. For the reasons mentioned above, we believe that the route of FMT should be tailored based on individual patient characteristics and clinical status. One study advocates using fidaxomicin and FMT to get maximum efficacy [13]. In a study that Alukal et al. published, the average number of days for symptom recovery was 4.62, with the earliest symptom reduction occurring two days after FMT [19].

Challenges involved in FMT include the availability of a healthy donor and the possibility of spreading infectious diseases if not screened properly [3]. One solution to this problem is the stool banks and the development of frozen capsulized preparations of stool [3,20]. FMT via colonoscopy may be slowly replaced by the oral consumption of laboratory-designed frozen oral capsulized preparations [3,8]. Another question is whether FMT is safe in immunocompromised patients, as is often the case in *C. difficile* patients. 

Limitations

In our systematic review, we could find very few randomized control trials as it would be difficult to conduct a trial in this vulnerable population group. Based on our inclusion/exclusion criteria, we did not include articles from other languages, involving animals, and studies published before 2012. In many of the studies we included, multiple confounding variables could have influenced the results as the population group of *C. difficile* infection often has multiple co-existing conditions. 

## Conclusions

This systematic review explored fecal microbiota transplantation as a treatment modality to decrease the morbidity and mortality related to *C. difficile* and its complications. Hospitalized elderly patients, especially in intensive care units and long-term care facilities, are the most commonly affected because of their multiple comorbidities, prolonged use of antibiotics and PPIs, poor gastrointestinal motility, and nosocomial exposure. Surgery as a treatment option for the complications of *C. difficile*, especially toxic megacolon, is highly risky considering the population mentioned above is often unfit for surgical management. There comes the role of FMT as a safe treatment modality that, in one or many sittings, can quickly resolve the patient’s symptoms and even prevent *C. difficile* related complications. The scope of FMT is not restricted to *C. difficile* infection and has been tried in several other gastrointestinal and neurological diseases. Questions remain regarding the route of administration of FMT, the timing of FMT, safety, availability, and the number of sittings required. More randomized controlled trials are necessary to address all these questions and to assess the safety of FMT. In our opinion, early use of FMT can substantially reduce *C. difficile* infection-related morbidity and mortality. 
